# The Complexity of Sequences Generated by the Arc-Fractal System

**DOI:** 10.1371/journal.pone.0117365

**Published:** 2015-02-20

**Authors:** Hoai Nguyen Huynh, Andri Pradana, Lock Yue Chew

**Affiliations:** 1 Complexity Institute, Nanyang Technological University, Singapore, Singapore; 2 Divison of Physics and Applied Physics, School of Physical and Mathematical Sciences, Nanyang Technological University, Singapore, Singapore; Universidad Veracruzana, MEXICO

## Abstract

We study properties of the symbolic sequences extracted from the fractals generated by the arc-fractal system introduced earlier by Huynh and Chew. The sequences consist of only a few symbols yet possess several nontrivial properties. First using an operator approach, we show that the sequences are not periodic, even though they are constructed from very simple rules. Second by employing the *ϵ*-machine approach developed by Crutchfield and Young, we measure the complexity and randomness of the sequences and show that they are indeed complex, i.e. neither periodic nor random, with the value of complexity measure being significant as compared to the known example of logistic map at the edge of chaos. The complexity and randomness of the sequences are then discussed in relation with the properties of associated fractal objects, such as their fractal dimension, symmetry and orientations of the arcs.

## Introduction

### 1.1 Literature review

Physical processes can, in general, be classified into three different categories, namely periodic (also quasi-periodic), random and chaotic processes. The first one refers to processes that are deterministic in nature and produce simple, regular and easily predictable patterns or observables (*e.g*. the motion of a simple pendulum or a spring-mass system); while the second one, as the name suggests, refers to processes that are stochastic in nature and produce complicated, irregular and unpredictable patterns (*e.g*. the classical coin toss experiment). The last category is somewhere in the middle between the former two. It refers to processes that can produce irregular patterns with some mixtures of predictability and unpredictability (*e.g*. chaotic pendulum system or the weather system). One can then ask a question of how to quantify the degree of regularity or predictability of a physical process. Equivalently, one asks: How complex is one process compared to another? To answer this question, it first has to be defined what is meant by “complex” and how to quantify it. Measure of complexity has long been investigated (*e.g*. the algorithmic (Kolmogorov-Chaitin) complexity [1–3], the Lempel-Ziv complexity [4], the logical depth of Bennett [5], the thermodynamic depth [6], the effective measure of complexity of Grassberger [7], the LMC complexity [8], the complexity measure by Shiner, Davison and Landsberg [9] *etc*…) and still remains actively explored in the recent years [10, 11]. In terms of defining complexity, one way is to relate it to randomness (*e.g*. the algorithmic (Kolmogorov-Chaitin) complexity [1–3]). Indeed, randomness is one aspect of complexity, in a sense that there is a “degree of randomness” or “degree of unpredictability” in the spectrum of these processes. Periodic processes are predictable, while random ones are maximally unpredictable.

The concept of randomness is crucial to complexity measure. However, it doesn’t capture the “statistical complexity” of a process. To illustrate this idea, consider the stochastic coin-flip experiment. It has high degree of randomness, but statistically speaking, it is simple. This statistical simplicity, or rather statistical complexity, is another way to define complexity for a physical process [12]. Building upon this perception, it can be seen that periodic and random processes should have low complexity as compared to chaotic processes. The high complexity of chaotic processes results from the fact that they are deterministic in nature yet produce irregular, unpredictable patterns. It is then interesting to study the complexity of physical processes or systems underlain by simple rules and understand how complexity arises from simplicity.

It is well known that many different systems capable of generating complicated patterns arise from simple rules, such as cellular automata or deterministic maps. Many features of such systems have been investigated, including orbit diagram and bifurcation map [13, 14]. Symbolic dynamics offers another interesting way to study the properties of the systems by assigning symbols, each associated to a state of the systems [15–18]. The corresponding symbolic sequence is then a representation of the systems as its dynamics is now encoded in the order of the symbols in the sequence. Such simple representation has become a convenient tool to study complexity of dynamical systems [11, 12, 19]. The study of complexity of dynamical systems, or rather their representative symbolic sequences, is not only interesting as argued in the paragraph above but also significant in terms of application within different fields, *e.g*. neurobehavioural or human pattern recognisation experiments. Such applications could be realised by mapping each symbol in a sequence to a corresponding state of a physical system and studying whether and how the human brain can perceive different states or even predict them [20, 21], *i.e*. the evolution of the system. It has also been shown that complexity theory is very relevant in the studies of different social organisations such as ant colonies [22] or business [23].

Having mentioned the context and significance of studying complexity of symbolic sequences, we notice that most of the studies so far deal with discrete maps or continuous dynamical systems, which are equation based, and therefore have no intrinsic nor genuine geometrical presentations. There is, on the other hand, a special class of systems, called fractals [24], that are also capable of generating complex patterns through geometrical operations. One prominent feature of fractal objects is their self-similarity which exhibits the degrees of predictability. However, the fine structure in fractals also introduces the unpredictability due to the existence of many different scales. It is therefore interesting to quantify how complex fractal objects are. In this article we employ the measure developed by Crutchfield and Young [12, 25] to explore the complexity of the sequences of symbols generated by a system called the arc-fractal system. Through this system, we introduce yet another way of generating complex symbolic sequences alongside the equation based methods of cellular automata or deterministic maps. This explicit way to extract (symbolic) sequences from fractal objects, which are geometrical objects, provides an intuitive link between the structure of the associated symbolic sequence and the spatial pattern of the object. To our best knowledge, such extraction has not been explored in the literature of fractals or complexity. And, hence, we expect our approach to be a rich source of complex sequences which can find useful applications.

In the next section, we introduce the arc-fractal system and how we can obtain the patterns of the fractals generated by the system and also discuss the properties of the symbolic sequences inferred from the patterns of the fractals. We then present the results on the complexity of these sequences. And finally, we conclude the study.

### 1.2 The arc-fractal system

#### 1.2.1 Idea of the arc-fractal system

**Fig 1:**
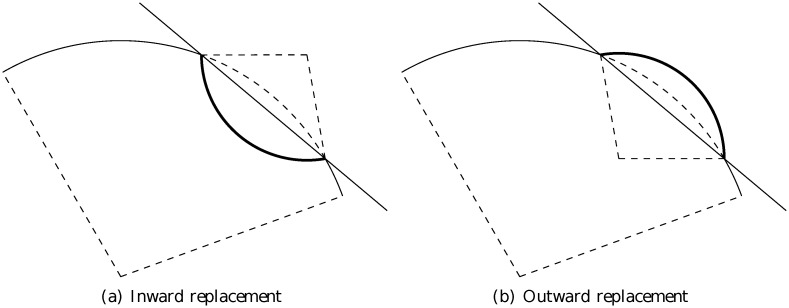
The replacement of an arc segment in the arc-fractal system. The line passing through the two end points of the arc segment is to determine the orientation of the new arc.

**Fig 2:**
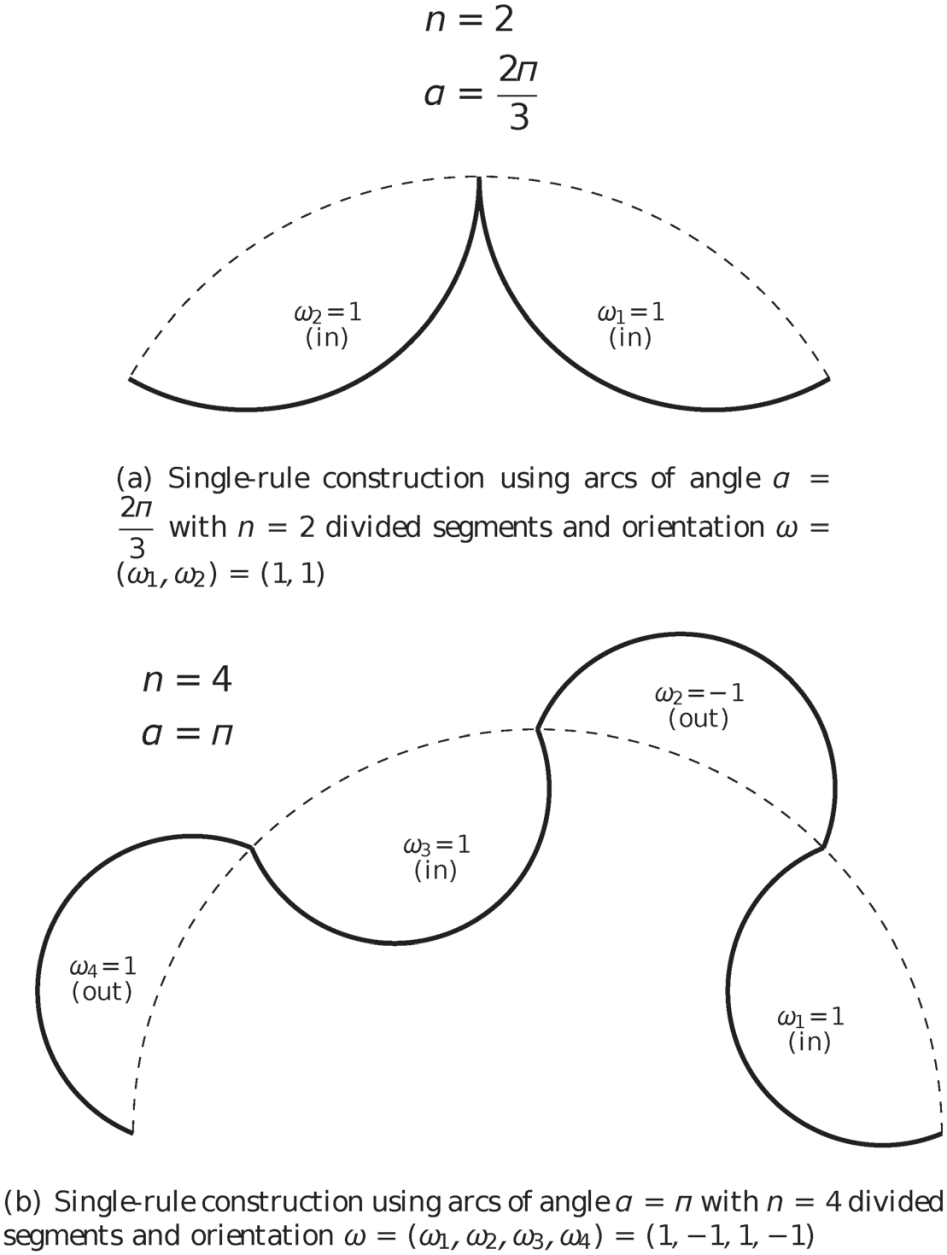
Illustration of the parameters *α*, *n* and *ω* in the construction of fractals using the arc-fractal system.

The arc-fractal system, which was introduced in [26, 27], is a simple yet universal fractal generator by applying recursive operations on geometrical arcs. At every step of iteration, every arc is divided into a number of segments and each segment is replaced by a new arc (see Fig. 1). There are three main parameters in the iteration process (see illustration in Fig. 2).

(a) *α*: the opening angle of the arc.
(b) *n*: the number of divided segments.
(c) ***ω***: the orientation of the replacing arcs (it is a vector of size *n* with elements *ω*_*i*_ being either 1 for inward arc or −1 for outward arc).

In general, there are three different types of rule for iteration in the arc-fractal system, namely single-rule, mutiple-rule and random-rule. Single-rule iteration means that the same set of values of the parameters (*α*, *n*, ***ω***) is used at every iteration (or level), including the initial arc being of the same angle *α*. Multiple-rule iteration means that different sets of values of the parameters (*α*, *n*, ***ω***) are employed at different iterations but follow a fixed pattern, *e.g*. set 1 applied at level 1, set 2 applied at level 2, set 3 applied at level 3 and the order repeats at higher levels. Random-rule iteration means that the sets of values of the parameters are different at different iterations and there is no pattern. In general, each parameter is a function of the level of iteration *m* and relative position *i* of the arc (with respect to the arc at the previous level that gives rise to it) to be replaced.

Normally, a feature of interest of a fractal object is its spatial pattern, *i.e*. self-similarity or fractal dimension. Here we introduce another interesting feature of the fractals generated by the arc-fractal system, that is the sequences of symbols associated wih the fractals. In what follow, we first introduce how the symbols can be extracted from the fractals and then discuss the relation between the symbols.

#### 1.2.2 Sequences from the arc-fractal system

As can be observed, at any iteration, the geometrical object generated by the arc-fractal system is a string of arcs of different orientations. (Note that the symbolic sequence generated at the infinite iteration is the one associated with the arc-fractal. That is, however, not achievable in practice.) If we associate each orientation of an arc element with a *unique* symbol, we can extract a sequence of symbols corresponding to that object. In this section, we shall describe the rules for labelling an arc element based on its orientation and also discuss on the basic features of the labels with respect to the arc-fractal system. The labelling rules were discussed in [26] and are summarised here for readability of the readers. These rules hold for the arc-fractal system with single-rule iteration, *i.e*. the same set of values of (*α*, *n*, ***ω***) is applied at every level, which is the main focus of our study. An example showing how to extract the sequences in the arrowhead and crab fractals is also provided.

**Fig 3:**
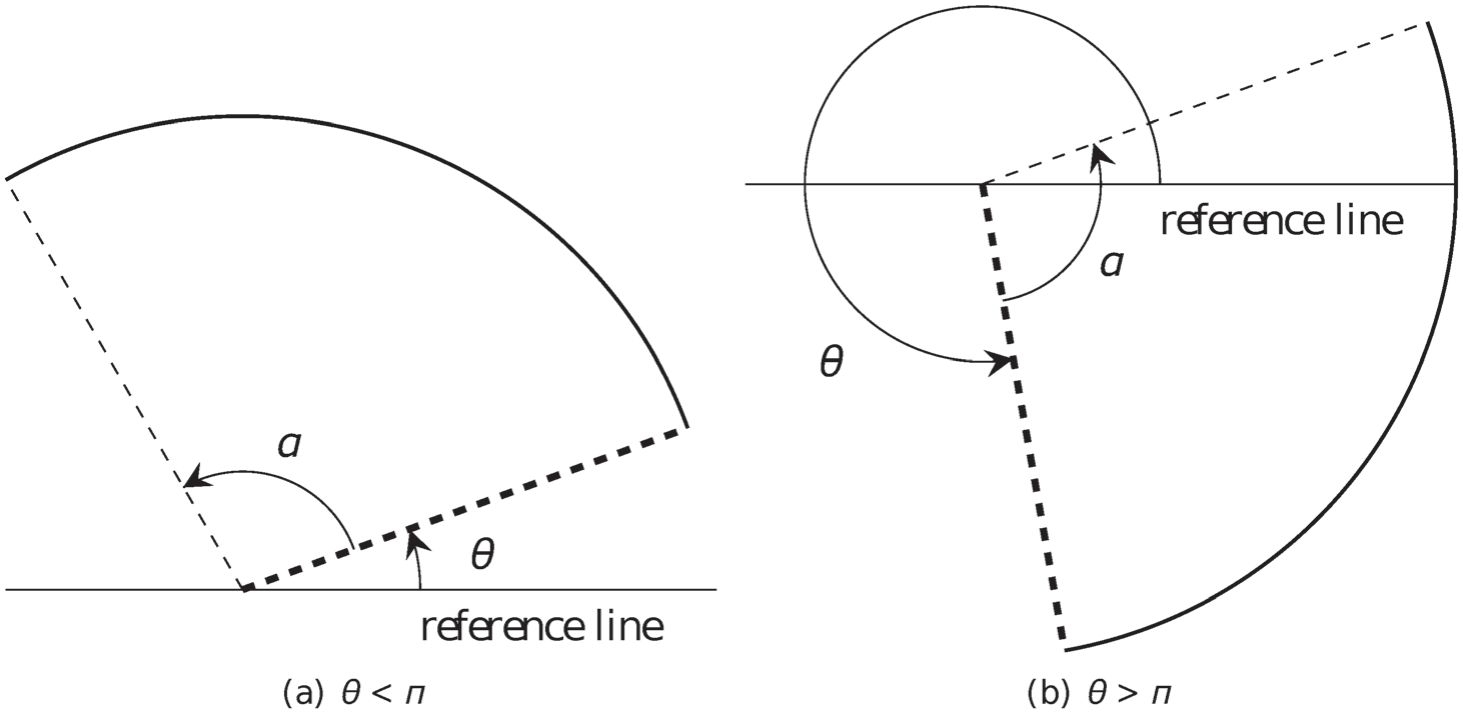
Definition of the angle *θ* of the orientation of an arc with a general opening angle *α*. An arc is drawn going counterclockwise from the thick dashed radial. *θ* is the (azimuth) angle made by the thick dashed radial and the (horizontal) reference line (see more in [27, p. 18]).

Throughout this work, for simplicity we only consider the case in which the angle of the arcs is *α* = *π*, *i.e*. the arcs are semicircles. In such case, it could be proven (see Eq. (A.19) in [27, p. 265]) that there are in total only 4*n* possible orientations associated with the arcs generated during the iteration process (however, at any level, there are at most only 2*n* orientations) or symbolically, there are 4*n* indices of rotation *I*. Each index *I* (which can take integer values between 1 and 4*n*) represents the orientation of an arc with azimuth angle *θ* (see its definition in Fig. 3) according to the relationship

$$\theta =\left(I-1\right)\frac{\pi }{2n},$$ (1)

with 0 ≤ *θ* < 2*π*. This equation says that the index *I* corresponds to a semicircle whose (radial) chord makes an angle of $(I-1)\frac{\pi }{2n}$ with the horizontal (or a reference line in general).

**Fig 4:**
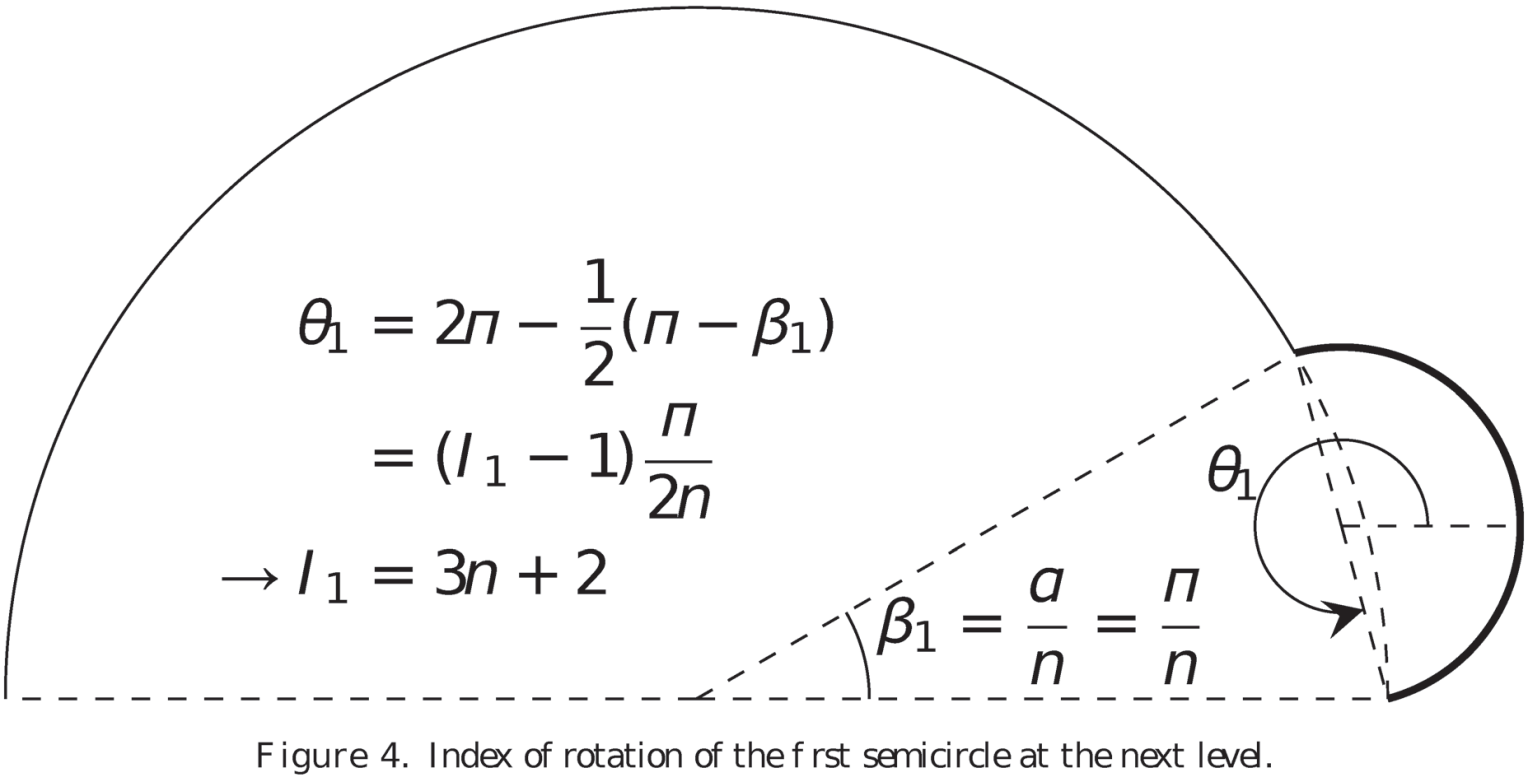
Index of rotation of the first semicircle at the next level.

In the construction of the fractals by the arc-fractal system, at every iteration, every arc is divided into *n* equal segments and each segment is replaced by a new arc. By knowing the index *I* of the original semicircle at level 0 (or the angle *θ* it makes with the reference line), we can obtain the indices of all the new semicircles that replace the *n* divided segments on the original semicircle. By some simple geometrical calculation illustrated in Fig. 4, given an initial arc of index *I* = 1 or *θ* = 0, we know that the first new semicircle at level 1 has index *I*_1_ = 3*n* + 2 if it is outward because the corresponding angle of orientation is $\theta_{1}=(3n+1)\frac{\pi }{2n}$. Otherwise, the semicircle has an index *I*_1_ = *n* + 2 if it is inward since, in which case, $\theta_{1}=(3n+1)\frac{\pi }{2n}+\pi -2\pi =(n+1)\frac{\pi }{2n}$. (Note that in our discussion, whenever necessary, there is an addition of (multiple of) 2*π* in the calculation to ensure that the angle *θ* is in the range [0, 2*π*). In other words, the addition of 2*π* is equivalent to taking the modulus of *I* with respect to 4*n*.) As could be easily seen, because of the symmetry of the semicircle, the inward arc replacement is simply a *π* rotation relative to the outward replacement. We then can go on to calculate the indices for all the remaning semicircles. The second one, which arises from the second segment, can be obtained via a counterclockwise rotation of $\frac{\pi }{n}$ with respect to the first semicircle. Therefore, it has an index of *I*_2_ = 3*n* + 4 if it is outward because $\theta_{2}=\theta_{1}+\frac{\pi }{n}=(3n+1)\frac{\pi }{2n}+\frac{\pi }{n}=(3n+3)\frac{\pi }{2n}$. Otherwise, with $\theta_{2}=(3n+3)\frac{\pi }{2n}+\pi -2\pi =(n+3)\frac{\pi }{2n}$, it has an index of *I*_2_ = *n* + 4 if it is inward. In this way, we can determine the indices of rotation of all the semicircles in the subsequent levels, which are summarised in Table 1.

**Table 1:** Indices of rotation of semicircles in the next level (arising from a semicircle of index *I* = 1).

**Index *I* of**	**First semicircle**	**Second semicircle**	***i*th semicircle**	***n*th semicircle**
Inward	*n* + 2	*n* + 4	*n* + 2*i*	3*n*
Outward	3*n* + 2	3*n* + 4	3*n* + 2*i*	*n*

In general, if the iteration process begins with a semicircle of some *I* other than *I* = 1, we can adjust the labelling by simply rotating the whole system counterclockwise by an angle of $(I-1)\frac{\pi }{2n}$. This is equivalent to increasing every index by an amount of *I* − 1, as illustrated in Table 2.

**Table 2:** Indices of rotation of semicircles in the next level (arising from a semicircle of general index *I*).

**Index *I* of**	**First semicircle**	**Second semicircle**	***i*th semicircle**	***n*th semicircle**
Inward	*I* + *n* + 1	*I* + *n* + 3	*I* + *n* + 2*i* − 1	*I* + 3*n* − 1
Outward	*I* + 3*n* + 1	*I* + 3*n* + 3	*I* + 3*n* + 2*i* − 1	*I* + *n* − 1

Once the indices of rotation of all the arcs have been determined, the corresponding sequence of symbols is simply a collection of indices as we string the arcs together in the counterclockwise direction (following the positive azimuth angle). It could be easily seen that the number of symbols in the sequence at level *m* is *n* ^*m*^ because at every iteration, one arc is replaced by *n* new arcs.

**Fig 5:**
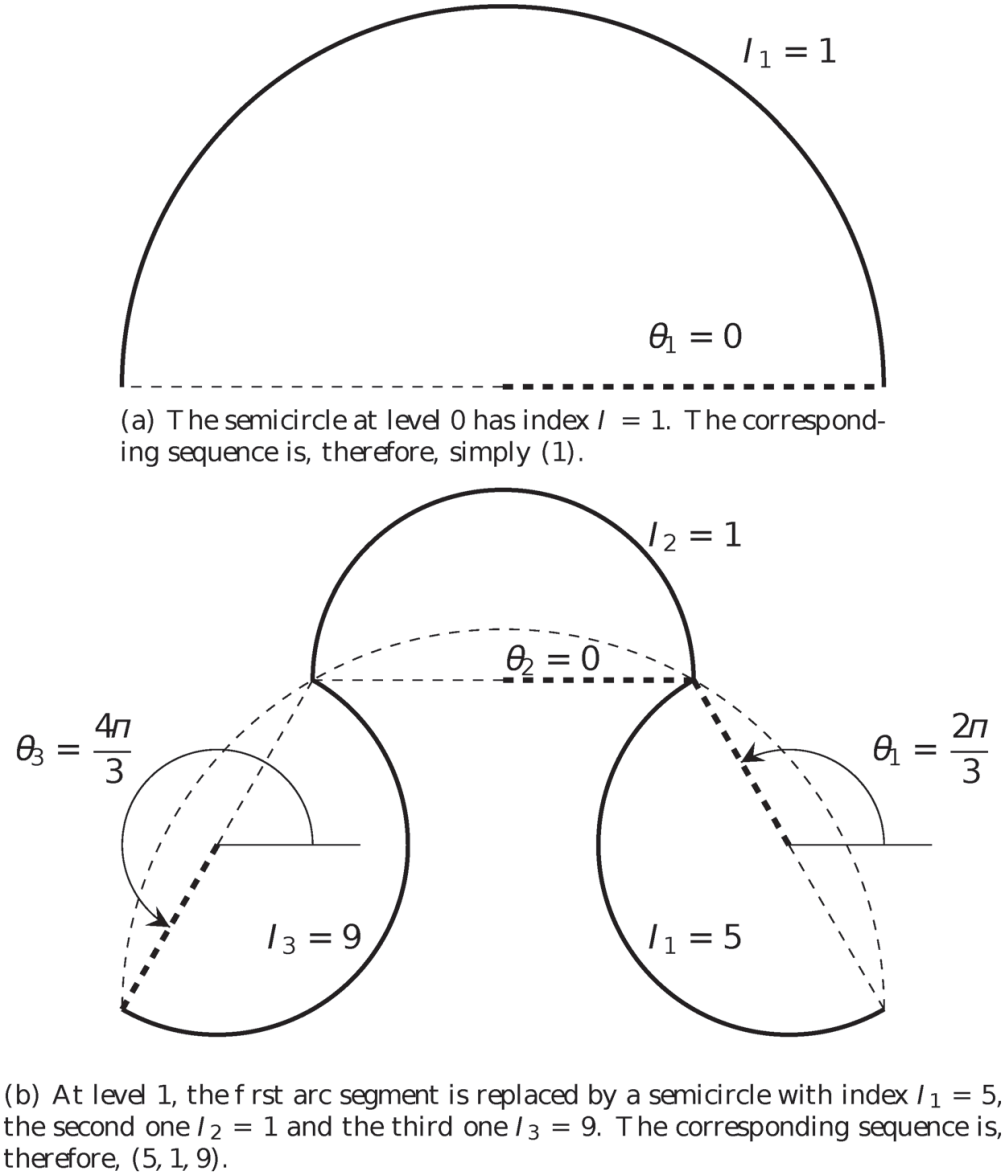
Sequences associated with the arrowhead fractal at different levels of construction. The rule is: *α* = *π*, *n* = 3 and ***ω*** = (1, −1, 1). The relation between the angle *θ*_*i*_ and the index *I*_*i*_ is given by Eq. (1).

**Fig 6:**
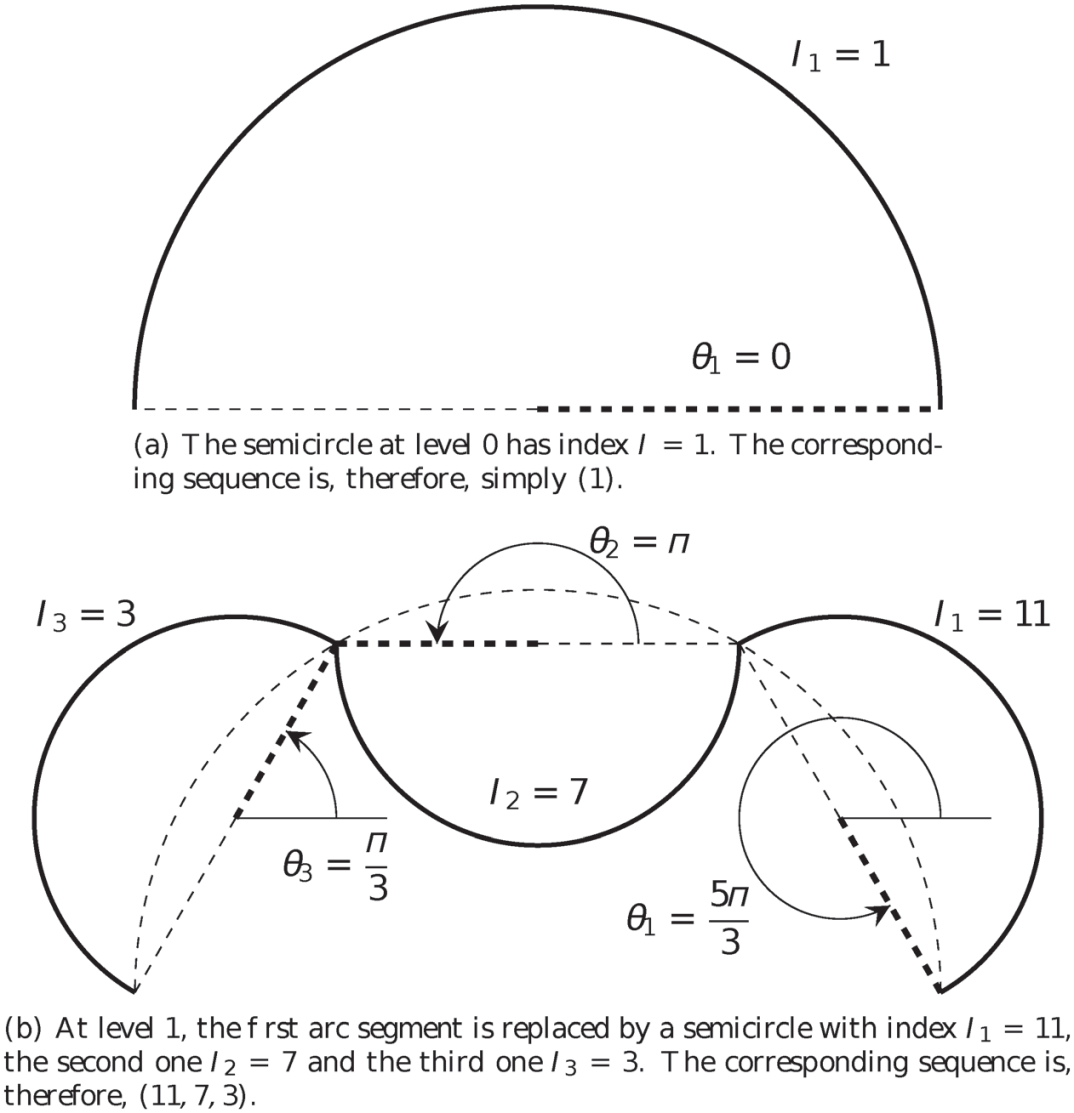
Sequences associated with the crab fractal at different levels of construction. The rule is: *α* = *π*, *n* = 3 and ***ω*** = (−1, 1, −1). The relation between the angle *θ*_*i*_ and the index *I*_*i*_ is given by Eq. (1).

As an example, the construction of the arrowhead and crab fractals and their associated sequences is illustrated in Figs. 5 and 6. We first consider the case of the arrowhead whose rule is *n* = 3 and ***ω*** = (1, −1, 1). Starting out with a semicircle at level 0, it could be easily seen that the corresponding sequence is (1). As discussed above, in the next level, the first new semicircle (Fig. 5(b), on the right) has index *I*_1_ = *n* + 2 = 5 because its orientation is inward making the angle $\theta_{1}=(n+1)\frac{\pi }{2n}=\frac{2\pi }{3}$. The second new semicircle (Fig. 5(b), at the middle) has index *I*_2_ = 3*n* + 4 = 1 (13 written as 1) because its orientation is outward making the angle $\theta_{2}=(3n+3)\frac{\pi }{2n}=0$ (2*π* written as 0). And the third new semicircle (Fig. 5(b), on the left) has index *I*_3_ = *n* + 6 = 9 because its orientation is inward making the angle $\theta_{3}=(n+5)\frac{\pi }{2n}=\frac{4\pi }{3}$. The corresponding sequence is, therefore, (5, 1, 9). Similarly, the sequence for the crab fractal at level 1 is (11, 7, 3) as shown in Fig. 6(b).

### 1.3 Properties of the arc-fractal sequences

In this section, we discuss some features of the arc-fractal system.

#### 1.3.1 Operators to generate the sequences

We observe that the arc-fractal constructed by the single-rule can be equivalently obtained by joining copies of the preceding level instead of replacing arc elements (see, for example, Eq. (7) below). This observation enables a mathematical construction of the sequence by employing two operators: rotation R and mirror inversion M which operate on a sequence of symbols associated with the arc-fractals [26, 27]. We denote $S^{m}=S_{1}^{m}S_{2}^{m}\ldots \;S_{n^{m}}^{m}$ as the sequence at a particular level *m* of construction. (In our notations, *S* ^*m*^ without the subscript denotes the entire sequence containing the symbols. $S_{i}^{m}$ with the subscript denotes the individual symbols in the sequence. As discussed at the end of Sec. 1.2.2, the size of the sequence at level *m* is *n* ^*m*^.)

The rotation operator R is then defined by

$$\begin{array}{cc} RS^{m} & =R\left(S_{1}^{m}\;S_{2}^{m}\;\ldots \;S_{n^{m}}^{m}\right)\\ & =S_{1}^{m}{}^{\prime }S_{2}^{m}{}^{\prime }\ldots \;S_{n^{m}}^{m}{}^{\prime }\\ & =S^{m}{}^{\prime }, \end{array}$$ (2)

where ${S_{i}^{m}}^{\prime }\equiv S_{i}^{m}+1\;\mathrm{mod}\;4n$ (*i* = 1, 2, …, *n* ^*m*^) (note that after the modulus, 0 is written as 4*n* as we adopt the convention that the indices take integer values between 1 and 4*n*). And the mirror inversion operator M is defined by

$$\begin{array}{cc} MS^{m} & =M\left(S_{1}^{m}S_{2}^{m}\ldots \;S_{n^{m}}^{m}\right)\\ & =S_{n^{m}}^{m}S_{n^{m}-1}^{m}\ldots \;S_{1}^{m}\\ & =\bar{S^{m}}. \end{array}$$ (3)

**Fig 7:**
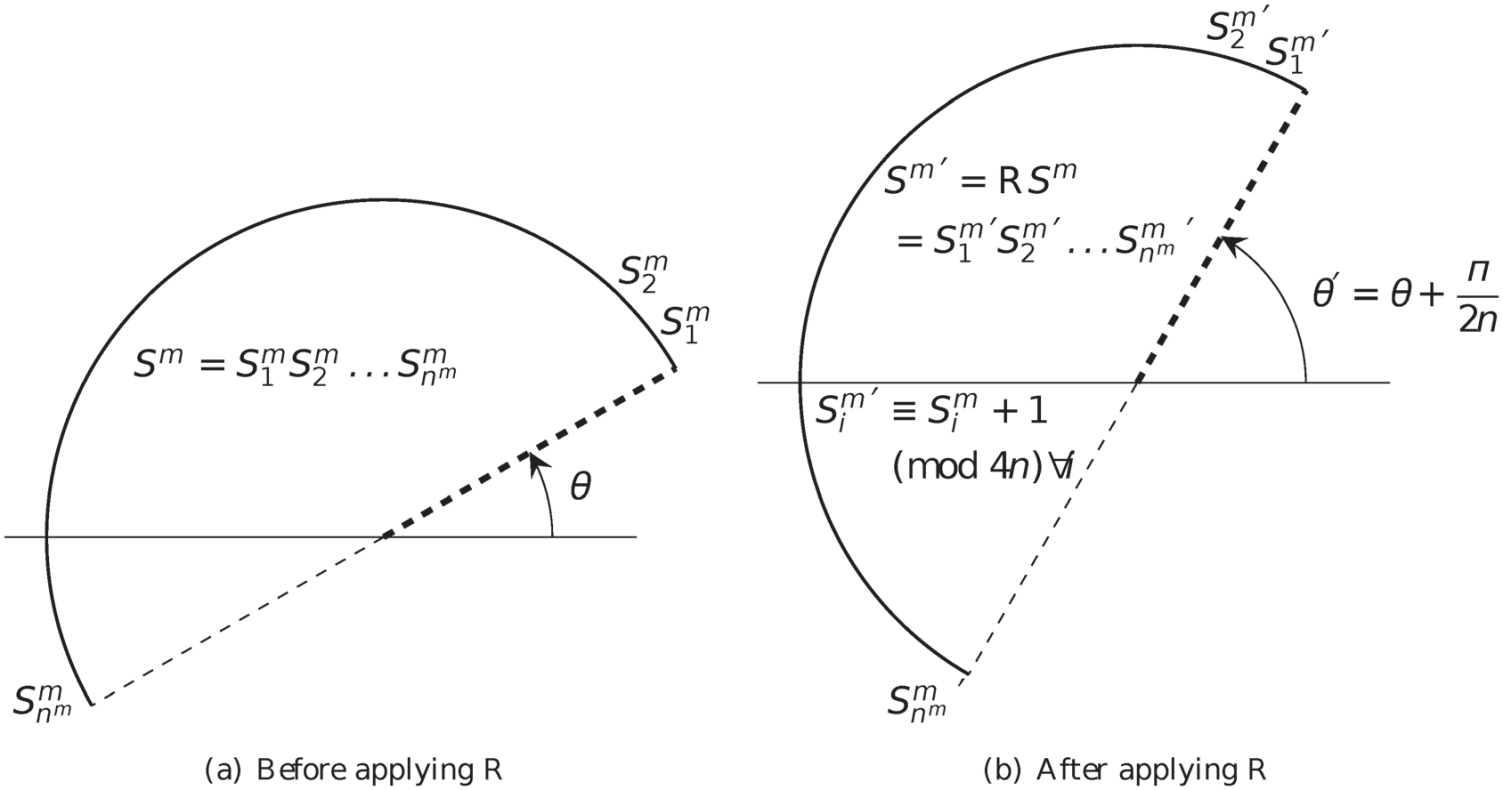
Action of the rotation operator R.

**Fig 8:**
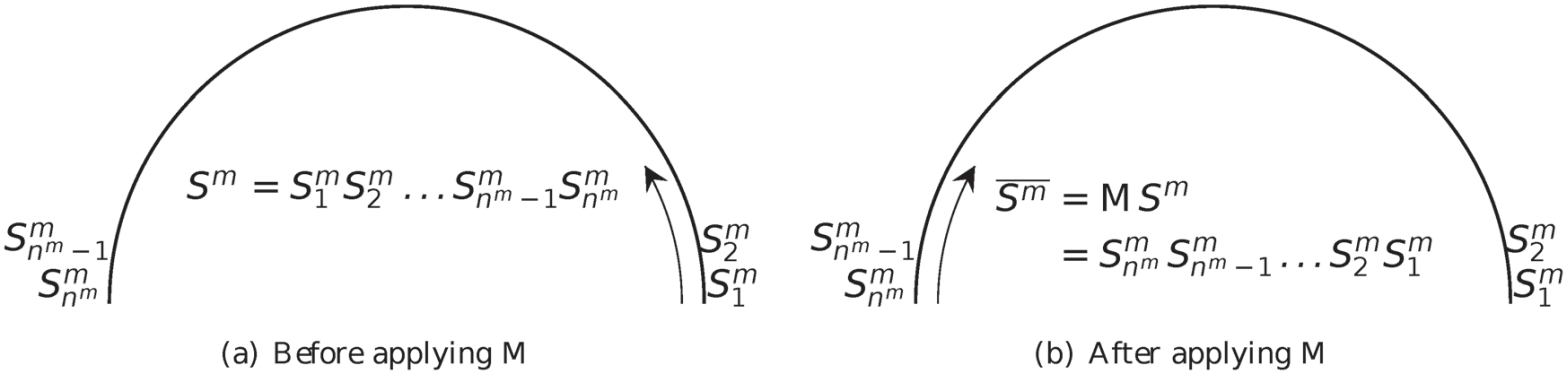
Action of the mirror operator M. The arrow indicates the order in which the labels are listed in the sequence.

Physically, operator R rotates the segment associated with sequence *S* ^*m*^ by an angle of $\frac{\pi }{2n}$ counterclockwise (see Fig. 7), whilst operator M does not make any change to the segment but reverses the order of the labels in the sequence (see Fig. 8). (Note that there is a slight modification here from the operator introduced in [26, 27], in which each application of R rotates the segment by $\frac{\pi }{n}$.) And it is clear that the two operators commute

MR=RM. (4)

In addition, the following identities hold

$$R^{4n}=I,$$ (5a)

$$M^{2}=I,$$ (5b)

where I is the identity operator $IS^{m}=S^{m}$. The identities in Eq. (5) simply mean that applying either the rotation operator R 4*n* times or the mirror inversion operator M two times, we get back the same sequence. Further discussion on the properties of the two operator can be found in [26, 27].

#### 1.3.2 Applying the operators to the arc-fractal sequences

Four different arc-fractal sequences are studied here and their construction in the operator form is given below. They are all constructed from semicircles, *i.e*.*α* = *π*, using single-rule, that is the same rule being applied at every iteration.

**Fig 9:**
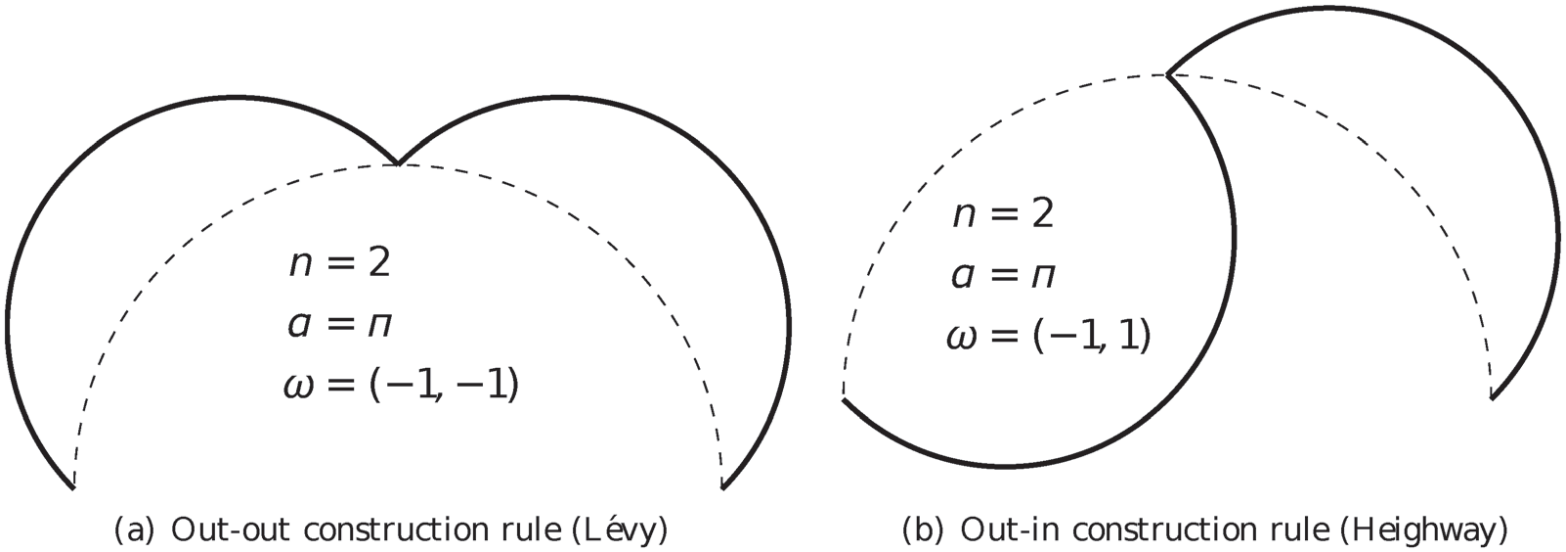
Construction rules of 2-arc fractals.

For Lévy fractal (see rule in Fig. 9(a)), the number of segments is *n* = 2 and the rule is “out-out”. According to Table 2, starting out with a semicircle of index *I* yields the first arc of index *I* + 3*n* + 1 = *I* + 7 and the second arc of index *I* + 3*n* + 3 = *I* + 9. Hence, given the sequence *S* ^*m*^ at level *m*, the sequence at next level *m* + 1 is given by

$$\begin{array}{cc} S^{m+1} & =(R^{7}S^{m})\;(R^{9}S^{m})\\ & =(R^{7}S^{m})\;(RS^{m}) \end{array}$$ (6)

because $R^{8}=I$ for *n* = 2. We may simply write Eq. (6) as $S^{m+1}=R^{7}S^{m}RS^{m}$ without the brackets with the convention that the operators R and M (or a combination of the two) only operate on the sequence following right after them. A pair of brackets is needed in order to indicate that the operators operate on the group of sequences enclosed in the brackets.

Similarly, for Heighway fractal (see rule in Fig. 9(b)), the number of segments is *n* = 2 and the rule is “out-in”, the equation is

$$S^{m+1}=R^{7}S^{m}MR^{5}S^{m}.$$ (7)

**Fig 10:**
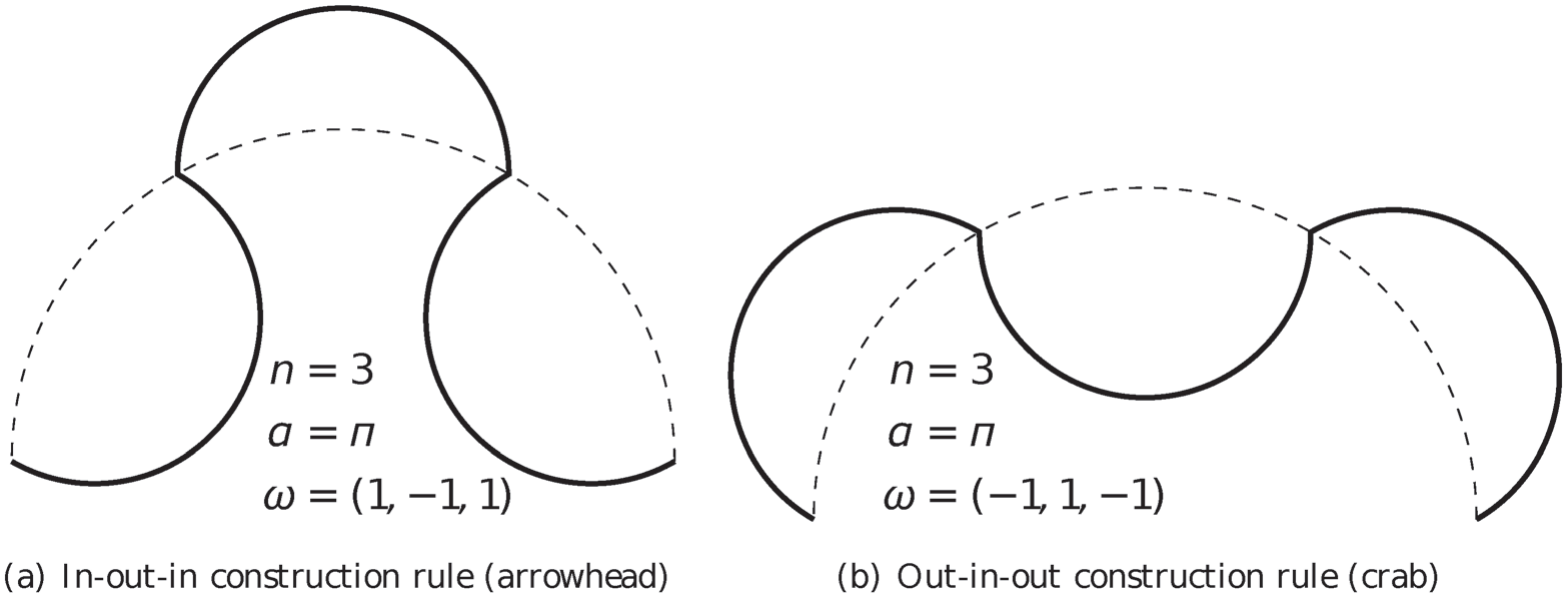
Construction rules of 3-arc fractals.

For arrowhead fractal (see rule in Fig. 10(a)), the number of segments is *n* = 3 and the rule is “in-out-in”, the equation is

$$S^{m+1}=MR^{4}S^{m}S^{m}MR^{8}S^{m}.$$ (8)

For crab fractal (see rule in Fig. 10(b)), the number of segments is *n* = 3 and the rule is “out-in-out”, the equation is

$$S^{m+1}=R^{10}S^{m}MR^{6}S^{m}R^{2}S^{m}.$$ (9)

Indeed, the general equation for an arc-fractal constructed from semicircles, *i.e*.*α* = *π*, using single-rule, is

$$S^{m+1}=A_{1}S^{m}A_{2}S^{m}\;\ldots \;A_{n}S^{m},$$ (10)

in which the operators $A_{i}$ are given by

$$A_{i}=M^{\frac{\omega_{i}+1}{2}}R^{(2-\omega_{i})n+2i-1}.$$ (11)

This equation comes about from the fact that the operator M is applied on the arc segments produced by inward replacement and the operator R is applied according to Table 2. As an illustration, we can recover Eq. (8) by substituting *n* = 3, *ω*_1_ = 1, *ω*_2_ = −1, *ω*_3_ = 1 into Eq. (11)

$$A_{1}=M^{\frac{1+1}{2}}R^{(2-1)\times 3+2\times 1-1}=MR^{4},$$ (12a)

$$A_{2}=M^{\frac{-1+1}{2}}R^{(2+1)\times 3+2\times 2-1}=R^{12}\equiv I,$$ (12b)

$$A_{3}=M^{\frac{1+1}{2}}R^{(2-1)\times 3+2\times 3-1}=MR^{8},$$ (12c)

and Eq. (10)

$$\begin{array}{cc} S^{m+1} & =A_{1}S^{m}A_{2}S^{m}A_{3}S^{m}\\ & =MR^{4}S^{m}IS^{m}MR^{8}S^{m}\\ & =MR^{4}S^{m}S^{m}MR^{8}S^{m}. \end{array}$$ (13)

In all Eqs. (6)–(10) above, we implicitly mean the concatenation of sequences by writing them side by side. For example, in Eq. (8), by writing $S^{m+1}=MR^{4}S^{m}S^{m}MR^{8}S^{m}$, we mean that the sequence *S* ^*m*+1^ is constructed by appending the sequence $MR^{8}S^{m}$ to the sequence *S* ^*m*^ and then the whole thing to the sequence $MR^{4}S^{m}$.

#### 1.3.3 Aperiodicity of the sequences

By definition, a periodic sequence is a sequence ${\left\{a_{i}\right\}}_{i=1}^{N}=a_{1},a_{2},\ldots ,a_{N}$ that satisfies

$$a_{i+p}=a_{i}$$ (14)

for all values of *i* and some value of *p*. In addition to that, it is also required that the size *N* of a periodic sequence ${\left\{a_{i}\right\}}_{i=1}^{N}$ must be a multiple of its period *p*. The reason for this second requirement is that a “basic” block of *p* successive entries must be repeated completely, *i.e*. every entry in the sequence must appear the same number of times as all the others.

Now we apply these criteria to the sequences associated with the four arc-fractal sequences introduced in Sec. 1.3.2 in order to show that they are not periodic. We have following theorem.

**Theorem.** *A sequence ${\left\{a_{i}\right\}}_{i=1}^{N}=a_{1},a_{2},\ldots ,a_{N}$ constructed using one of the rules given by Eqs*. (6)–(9) *above is not a periodic sequence*.

**Proof.** From the definition in Eq. (14) above, the general strategy to prove the aperiodicity of a sequence is simply to show that there exists *at least* one pair of entries *a*_*i*_ and *a*_*i*+*p*_ that are not the same for *any* value of period *p*, which is a divisor of *N*. This strategy is applied to all sequences considered in this study.

*a. Crab sequence*

We denote $S_{i}^{m}$ as the *i*th entry in the sequence at level *m*. At level *m*, the size of the sequence is *N* = 3^*m*^. Since 3 is a prime number, the period *p* can only take the values *p*_*k*_ = 3^*k*^ (*k* = 0, …, *m* − 1). Since the condition in Eq. (14) has to be fulfilled for *all* values of *i*, it is sufficient to prove that ${\left\{S_{i}^{m}\right\}}_{i=1}^{3^{m}}$ is *not* periodic by showing that $S_{i+p}^{m}\neq S_{i}^{m}$ for *some* value of *i*. It is easy to see that for a sequence of size 3^*m*^, showing that $S_{i}^{m}\neq S_{i+p_{m-1}}^{m}$ is sufficient to prove that the sequence is not periodic for all periods *p*_*k*_ = 3^*k*^ (*k* = 0, …, *m* − 1). This is because $S_{i}^{m}$ and $S_{i+p_{m-1}}^{m}$ are at the same relative position when any period *p*_*k*_ is considered.

From Eq. (9) we see that the first entry of the sequence at level *m* is given by $S_{1}^{m}={\left({\text{R}}^{10}\right)}^{m}\;C^{0}$ where *C* ^0^ is the number associated with the initial arc at level 0. It is an observation that, at level *m*, the entry $S_{1+3^{m-1}}^{m}$ is determined by the first entry of the second part of the crab fractal at the same level. Because of the operator M on the second part during the construction, this first entry is indeed determined by the last entry of the first part of the crab fractal at the same level. From the same Eq. (9), we know that the last entry of the crab fractal at level *m* − 1 is given by $S_{3^{m-1}}^{m-1}={\left({\text{R}}^{2}\right)}^{m-1}\;C^{0}$. And therefore, the entry $S_{1+3^{m-1}}^{m}$ is given by $R^{6}{\left(R^{2}\right)}^{m-1}\;C^{0}$. Now we compare the two entries $S_{1}^{m}={\left({\text{R}}^{10}\right)}^{m}\;C^{0}=C^{0}+10m$ and $S_{1+3^{m-1}}^{m}={\text{R}}^{6}{\left({\text{R}}^{2}\right)}^{m-1}\;C^{0}=C^{0}+6+2(m-1)=C^{0}+2m+4$ of the sequence at level *m*. We recall from Sec. 1.2.2 that we take the modulus of 4*n* for every entry in the sequence (after which 0 is written as 4*n*). In the case of crab sequence, we take modulus 12 and we realise that the entries $S_{1}^{m}$ and $S_{1+3^{m-1}}^{m}$ can be equal for *m* fulfilling the condition

$$C^{0}+10m\equiv C^{0}+2m+4\;\mathrm{mod}\;12$$ (15)

or

8m+8≡0mod12. (16)

Even in the event that Eq. (16) is satisfied, we still can prove that the sequence is not periodic by showing that other pairs of $S_{i}^{m}$ and $S_{i+p}^{m}$ do not match for the *same m*. One such pair comprises of $S_{3^{m-1}}^{m}$ and $S_{2\times 3^{m-1}}^{m}$. The reason for this choice is that $S_{3^{m-1}}^{m}$ is determined by the last entry of the sequence at level *m* − 1, $S_{3^{m-1}}^{m-1}$, whilst $S_{2\times 3^{m-1}}^{m}$ is determined by the first entry of the sequence at the same level, $S_{1}^{m-1}$. Using Eq. (9), we have $S_{3^{m-1}}^{m}={\text{R}}^{10}{\left({\text{R}}^{2}\right)}^{m-1}\;C^{0}=C^{0}+10+2(m-1)=C^{0}+2m+8$ and $S_{2\times 3^{m-1}}^{m}={\text{R}}^{6}{\left({\text{R}}^{10}\right)}^{m-1}\;C^{0}=C^{0}+6+10(m-1)=C^{0}+10m-4$. Again, we take modulus 12 and the entries $S_{3^{m-1}}^{m}$ and $S_{2\times 3^{m-1}}^{m}$ can be equal for *m* fulfilling the condition

$$C^{0}+2m+8\equiv C^{0}+10m-4\;\mathrm{mod}\;12$$ (17)

or

8m≡0mod12. (18)

It is straightforward to see that Eqs. (16) and (18) cannot be satisfied for the *same* value of *m*, i.e. when $S_{1}^{m}=S_{1+3^{m-1}}^{m}$ we have $S_{3^{m-1}}^{m}\neq S_{2\times 3^{m-1}}^{m}$ and vice versa when $S_{3^{m-1}}^{m}=S_{2\times 3^{m-1}}^{m}$ we have $S_{1}^{m}\neq S_{1+3^{m-1}}^{m}$. That means the sequence is not periodic.

*b. Arrowhead sequence*

For this sequence, the situation is more complicated than for the crab as the first part and the last part are reverse. From Eq. (8), we see that because of the operator M on the first part, the first entry of the sequence at level *m*, $S_{1}^{m}$ is determined by the last entry of the sequence at the previous level *m* − 1, $S_{3^{m-1}}^{m-1}$. Similarly, because of the operator M on the last part, the last entry of the sequence at level *m*, $S_{3^{m}}^{m}$ is determined by the first entry of the sequence at the previous level *m* − 1, $S_{1}^{m-1}$. We can construct Table 3 to determine the first and last entry of the arrowhead sequence at different levels, using Eq. (8).

**Table 3:** First and last entry of the arrowhead sequence at different levels.

**Level *m***	**First entry $S_{1}^{m}$**	**Last entry $S_{3^{m}}^{m}$**
0	*C* ^0^	*C* ^0^
1	${ℜ}^{4}C^{0}$	${ℜ}^{8}C^{0}$
2	${ℜ}^{4}{ℜ}^{8}C^{0}$	${ℜ}^{8}{ℜ}^{4}C^{0}$
3	${ℜ}^{4}{ℜ}^{8}{ℜ}^{4}C^{0}$	${ℜ}^{8}{ℜ}^{4}{ℜ}^{8}C^{0}$
4	${ℜ}^{4}{ℜ}^{8}{ℜ}^{4}{ℜ}^{8}C^{0}$	${ℜ}^{8}{ℜ}^{4}{ℜ}^{8}{ℜ}^{4}C^{0}$

We have the following relations

$$S_{1}^{m}=R^{4}S_{3^{m-1}}^{m-1},$$ (19a)

$$S_{3^{m}}^{m}=R^{8}S_{1}^{m-1}.$$ (19b)

It reveals that we have to consider two different cases for *m* being odd and even. At even level *m* = 2*t*, the first entry is given by

$$\begin{array}{cc} S_{1}^{m} & ={\left(R^{4}\right)}^{t}\;{\left(R^{8}\right)}^{t}\;C^{0}\\ & ={\left(R^{4}R^{8}\right)}^{t}\;C^{0}\\ & ={\left(R^{12}\right)}^{t}\;C^{0}\\ & =I^{t}C^{0}\\ & =C^{0}. \end{array}$$ (20)

The last entry is also given by the same value $S_{3^{m}}^{m}=C^{0}$. Using Eq. (8), we have $S_{1+3^{m-1}}^{m}={\text{R}}^{4}C^{0}=C^{0}+4$, i.e. the first entry at the previous level which is an odd level (refer to Table 3). From there, it is clear that $S_{1}^{m}\neq S_{1+3^{m-1}}^{m}$. Similarly, at odd level *m* = 2*t* + 1, the first entry is given by $S_{1}^{m}={\text{R}}^{4}C^{0}$ and the last entry is given by $S_{3^{m}}^{m}={\text{R}}^{8}C^{0}$. We have $S_{1+3^{m-1}}^{m}=C^{0}$, i.e. the first entry at the previous level which is an even level. From there, it is clear that $S_{1}^{m}\neq S_{1+3^{m-1}}^{m}$. So for either even or odd *m*, the arrowhead sequence is not periodic for any period.

*c. Lévy sequence*

From Eq. (6) we have the first and last entries given by

$$S_{1}^{m}={\left(R^{7}\right)}^{m}\;C^{0},$$ (21a)

$$S_{2^{m}}^{m}=R^{m}C^{0}.$$ (21b)

Hence,

$$\begin{array}{cc} S_{1+2^{m}}^{m+1} & =RS_{1}^{m}\\ & =RR^{7m}C^{0}\\ & =C^{0}+7m+1 \end{array}$$ (22)

and it is clear that $S_{1}^{m}=C^{0}+7m\neq S_{1+2^{m-1}}^{m}=C^{0}+7m-6$ for all values of *m*. Therefore, the Lévy sequence is not periodic for any period at any level.

*d. Heighway sequence*

From Eq. (7) we have the first and last entries given by

$$S_{1}^{m}={\left(R^{7}\right)}^{m}\;C^{0},$$ (23a)

$$S_{2^{m}}^{m}=R^{5}S_{1}^{m-1}=R^{5}{\left(R^{7}\right)}^{m-1}\;C_{0}.$$ (23b)

Hence

$$\begin{array}{cc} S_{1+2^{m}}^{m+1} & =R^{5}S_{2^{m}}^{m}\\ & =R^{5}R^{5}{\left(R^{7}\right)}^{m-1}\;C^{0}\\ & =C^{0}+7m+3 \end{array}$$ (24)

and it is clear that $S_{1}^{m}=C^{0}+7m\neq S_{1+2^{m-1}}^{m}=C^{0}+7m-4$ for all values of *m*. Therefore, the Heighway sequence is not periodic for any period at any level.

## Methods

We know that the sequences associated with the arc-fractals above are deterministic because the rules to construct the fractals are deterministic, i.e. they are not random. On the the other hand, it has also been shown in Sec. 1.3.3 that all the sequences are not periodic. From the perspective of complexity, we know that in between periodicity and complete randomness, there lies a wide spectrum which is said to be complex. In this section, we will show that the arc-fractal sequences are indeed complex by considering their measures of complexity. In this work, there are two important measures that we are going to use to quantify the degree of randomness and complexity of a physical process.

### 2.1 Information theory and randomness

The first quantity is *metric entropy* which quantifies the degree of randomness of a physical process. This quantification of the degree of randomness is based on the idea from information theory, which was developed by Shannon [28] in 1940s and formed the basis of modern theory of communication. For a sequence of symbols ${\left\{a_{i}\right\}}_{i=1}^{N}=a_{1},a_{2},\ldots ,a_{N}$, its information content or Shannon entropy is given by

$$H(N)=-\sum_{\left\{a_{i}\right\}}[P(a_{1},a_{2},\ldots ,a_{N})\times \log_{2}P(a_{1},a_{2},\ldots ,a_{N})]\text{,}$$ (25)

in which *P*(*a*_1_, *a*_2_, …, *a*_*N*_) is the probability of occurrence of a particular sequence (*a*_1_, *a*_2_, …, *a*_*N*_). The unit is *bit* when using base-2 logarithm.

The *metric entropy* was developed by Kolmogorov [1] and Sinai [29]. It is the rate (per symbol) of the Shannon entropy and is mathematically given by $h=\underset{N\to \infty }{\lim}\frac{H(N)}{N}$. In practice, it is impossible to obtain an infinite sequence of measurement from any real system but one can still make the approximation

$$h\approx \frac{H(N)}{N}$$ (26)

for sufficiently large *N*. In some situations, it is more useful to calculate the linear fit of the slope

$$h=\frac{H\left(N_{2}\right)-H\left(N_{1}\right)}{N_{2}-N_{1}}$$ (27)

when *N* is not large enough. Regarding each measurement as a symbol in the sequence, metric entropy could then be viewed as the information gain per symbol and therefore its unit is *bit*/*symbol*.

In application to the arc-fractal sequences, this metric entropy as a measure of randomness tells us how “random” a sequence of arcs is. As each arc is translated to a symbol via its orientation of rotation, the entire sequence encapsulates the orientations of the arcs and hence the geometrical shape of the object constituted by the arcs. Such measure of randomness might provide us with another quantity to characterise the structure of the (fractal) object beside its (fractal) dimension, e.g. a periodic sequence indicates a simple string of arcs with regularly repeated orientations.

### 2.2 Statistical complexity

The second quantity we would use to measure the complexity of a physical process is called *statistical complexity*. In the definition of statistical complexity, a representation to model physical systems called the *ε*-machine is employed. Constructing this representation involves grouping historical events into *causal states* together with determining transitions among them [12].

Given the causal states *σ*’s of an *ε*-machine and their probabilities *P*_*σ*_’s, the statistical complexity of the physical process modelled by this machine can be computed directly by

$$C_{S}=-\sum_{\sigma }P_{\sigma }\log_{2}P_{\sigma },$$ (28)

whose unit is *bit*. As already discussed by Crutchfield and Young [12, 25], this statistical complexity measures the statistical property of a system. More insight into the concept of statistical complexity is discussed in details by Crutchfield in [25].

These two measures of *metric entropy* and *statistical complexity* are complementary to each other. In the context of patterns, the measure of randomness is easy to perceive. It entails the lack of predictability or lack of patterns in a process, i.e. the higher the randomness of the process is, the lesser the patterns it posseses. On the other hand, the statistical complexity measure describes how complex the “rules” that the process follows are, in the sense that it measures the “mixture” or intercorrelation between randomness and patterns in a process. It is, therefore, useful to use the measure of randomness in conjunction with the statistical complexity measure to gain a more complete picture of a system.

In the context of the current work on the arc-fractal sequences, the *ε*-machine complexity is readily applicable to the sequences, since the representation of the orientation of the arcs in the arc-fractals is in the form of sequence of symbols, consistent with the requirement of the *ε*-machine complexity that a physical process is described in a sequence of symbols.

Another compelling feature of the *ε*-machine is that it has a clear physical picture attributed to it, which is that of the simplest representative machine, consisting of states and transitions among the states, that can generate outcome with the same statistical properties as the original sequence. Furthermore, the formulation of the complexity of the *ε*-machine follows the familiar statistical entropic formulation on the machine states’ probability distribution (Eq. (28)).

In the arc-fractal generation process, there is no variable parameter that can be tuned continuously. Instead, what can be varied are the rules of construction. As we can see in the previous section, by varying even a small part of the rules, the resulting arc-fractal can be quite different. Since the *ε*-machine concerns the generation of the outcome of the process, the *ε*-machine complexity can indeed be considered as the complexity of the generation process itself. Using the *ε*-machine, we want to see how the complexity of the arc-fractal generation process changes by changing the rules of its construction.

## Results

Having described the two measures, we now move on to applying them to the sequences from the arc-fractals introduced earlier. We start by considering the sequence associated with the crab fractal (rule described in Fig. 10(b)) at level 3. Using Eq. (9), we can construct this sequence starting from an arc of index *C*_0_ = 1 at level 0. We have

$$S^{0}=C_{0}=1$$ (29a)

$$\begin{array}{cc} S^{1} & =\underline{R^{10}S^{0}}\;\underline{MR^{6}S^{0}}\;\underline{R^{2}S^{0}}\\ & =\underline{11},\underline{7},\underline{3} \end{array}$$ (29b)

$$\begin{array}{cc} S^{2} & =\underline{R^{10}S^{1}}\;\underline{MR^{6}S^{1}}\;\underline{R^{2}S^{1}}\\ & =\underline{9,5,1},\underline{9,1,5},\underline{1,9,5} \end{array}$$ (29c)

$$\begin{array}{cc} S^{3} & =\underline{R^{10}S^{2}}\;\underline{MR^{6}S^{2}}\;\underline{R^{2}S^{2}}\\ & =\underline{7,3,11,7,11,3,11,7,3},\\ & \;\underline{11,3,7,11,7,3,7,11,3},\\ & \;\underline{11,7,3,11,3,7,3,11,7} \end{array}$$ (29d)

The underlines group the symbols according to the operators for easy tracking. It is observed that the sequence

$$\begin{array}{c} (7,3,11,7,11,3,11,7,3,\\ 11,3,7,11,7,3,7,11,3,\\ 11,7,3,11,3,7,3,11,7) \end{array}$$

contains only three different types of symbol 3, 7 and 11. Hence, it is then converted to a base-3 sequence for simplicity in the computation of the measures

$$\begin{array}{c} (1,0,2,1,2,0,2,1,0,\\ 2,0,1,2,1,0,1,2,0,\\ 2,1,0,2,0,1,0,2,1). \end{array}$$

**Fig 11:**
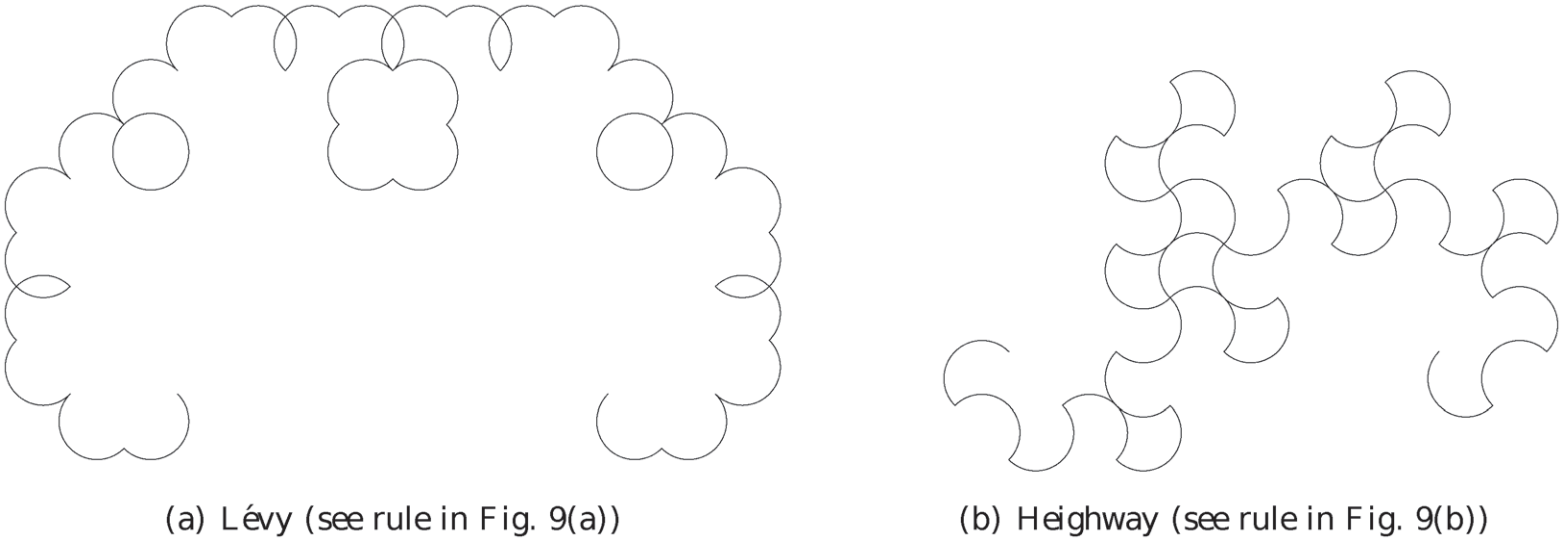
2-arc fractals at level 5.

**Fig 12:**
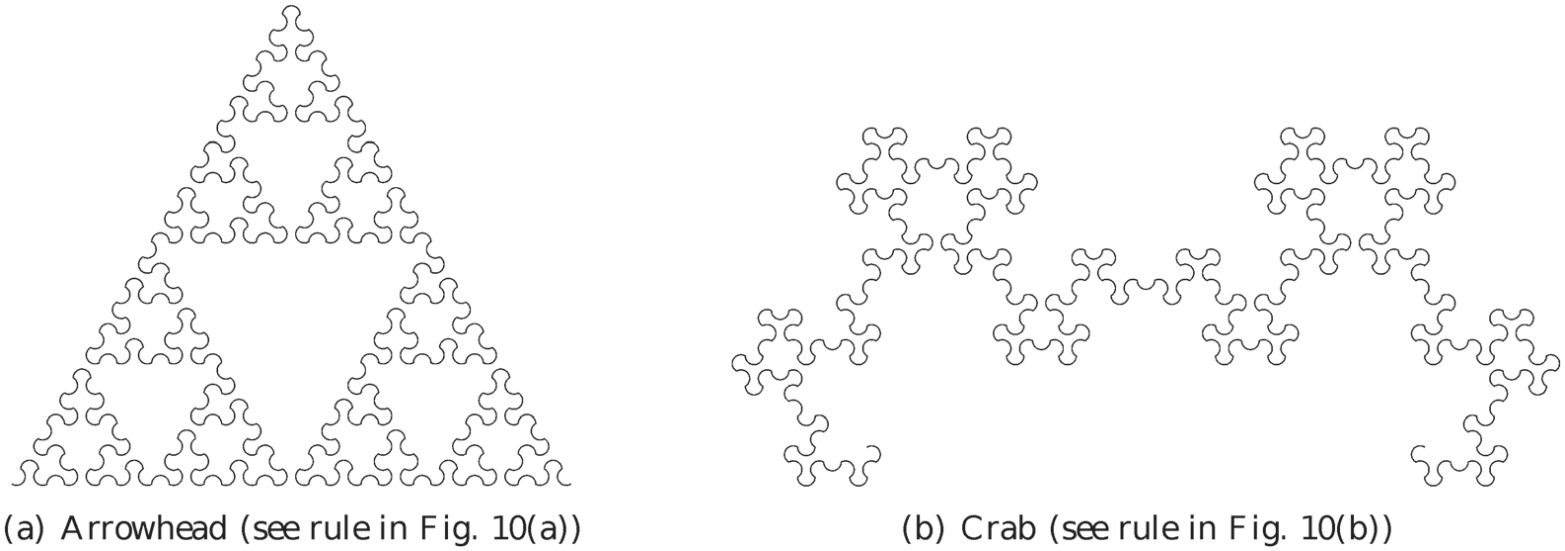
3-arc fractals at level 5.

Figs. 11 and 12 show the four arc-fractals considered in this study at level 5, for rules involving 2 arcs (out-in (Heighway) and out-out (Lévy)) and 3 arcs (out-in-out (crab) and in-out-in (arrowhead)) respectively.

Before the results are presented, it is important to note that it is practically impossible to create arc-fractals with level of construction *m* approaching infinity. Fortunately, our results show that for the arc-fractal sequence, the values of metric entropy and complexity are asymptotic at the higher levels; i.e. they approach certain values at very high levels of iteration.

Table 4 shows the main results for different types of arc-fractal sequences at higher levels. The entropy and statistical complexity values in the table were computed using Eq. (26) and Eq. (28) correspondingly.

**Table 4:** Results of arc-fractal sequences with 2 arcs (Heighway and Lévy) and 3 arcs (arrowhead and crab).

**Fractal**	**dimension *d***	**number of arcs *n***	**orientations of arcs**	**level *m***	**length *n*^*m*^**	**number of symbols^†^**	***N***	***h* (bit/symbol)**	***C*_*S*_ (bit)**
Lévy	2	2	out-out	27	2^27^	4	20	0.389	9.80
Heighway	2	2	out-in	27	2^27^	4	20	0.359	9.27
Arrowhead	$\frac{\ln3}{\ln2}$	3	in-out-in	17	3^17^	3	20	0.387	7.34
Crab	$\frac{\ln3}{\ln2}$	3	out-in-out	17	3^17^	3	20	0.387	7.34

^†^ Even though it has been discussed in the text in Sec. 1.2.2 and shown in [27, p. 265], it turns out that at a particular level, the sequences for arc-fractal with even *n* have 2*n* types of symbols while those with odd *n* have *n* types of symbols.

We computed the results using the arc-fractal sequences of level 27 for 2-arc (Heighway and Lévy) and level 17 for 3-arc (arrowhead and crab) fractals. For the *ε*-machines of all the arc-fractal types, the underlying tree was constructed from 40-cylinders and machine from 20-cylinders. (Note that the machine cylinder number is basically the same as sequence (or symbol) length *N*. Larger tree cylinder number is required for sub-tree comparison purpose. See [30] for more details.)

It should be noted that calculating the metric entropy using Eq. (26) and Eq. (27) would yield different results. Either equation is generally acceptable for the purpose of comparison among the arc-fractal types, as long as one is consistent with the choice of equation. Furthermore, we chose Eq. (26) to be consistent with [12].

Our computational method follows the algorithm presented in [30] with modifications. We checked the validity of our method by reproducing the results of the complexity measures of the logistic map in [12].

The choice of the levels of the arc-fractal is determined by the required computational memory. We also note that, as discussed before, the values of metric entropy and complexity asymptotically approach certain values at higher levels. Our choice of levels of construction (level 27 for the 2-arc and level 17 for the 3-arc fractals) is good enough based on the fact that the values of both the complexity measure and metric entroy converge to asymptotic values with very small standard deviations (compared to the values of the measures themselves in Table 4) being less than 5 × 10^−2^ *bit* and 10^−5^ *bit*/*symbol* respectively for all the sequences considered.

The level of construction would determine the total length of the arc-fractal sequences. After choosing the level of construction of an arc-fractal type, one has to determine the suitable choice of length of machine cylinder number (the same as sequence (or symbol) length *N*) and tree cylinder number. The tree cylinder number (or tree length *L*_*T*_), being the larger between the two cylinder numbers, should be such that when a set is constructed containing all the contiguous sub-sequences of length *L*_*T*_ of the arc-fractal sequence, the set has to contain every possible combination of *L*_*T*_-sequences that can be generated by the arc-fractal generation process. With an infinite sequence (or infinite level of construction), we can choose any arbitrary tree cylinder number. However, with a finite sequence, the tree cylinder number is limited. Simply said, the tree cylinder number *L*_*T*_ is limited by the total length of the arc-fractal sequence. The machine cylinder number *N*, in a sense, characterises the range of the construction of the *ε*-machine (hence machine cylinder). Larger *N* is better to capture a more complete *ε*-machine. However, it is limited by the tree cylinder number *L*_*T*_, since *L*_*T*_ > *N*. Since *L*_*T*_ is required for sub-tree comparison purpose, it is preferable for *L*_*T*_ to be reasonably larger than *N* for better sub-tree comparison. Here, we choose $N=\frac{1}{2}L_{T}$. We note that our choice of parameters is also determined by what our computational resources can manage.

In general, it is not necessary to determine the level of construction (or the length of sequence) first, and then determine the other parameters. The choice of parameters should take into consideration the limitations one parameter has on the others, balanced by the computational resources.

## Discussion

It could be easily shown that for a base-*b* pseudo-random sequence (having *b* different types of symbol that are uniformly distributed among each other), its maximum value of entropy is *h* = log_2_ *b*. Compared to that, the metric entropies for the arc-fractal sequences discussed are much smaller in value. In terms of randomness, we can say to some extent that the sequences from the arc-fractal system are not that random. In other words, the construction of the arc-fractals does not produce sufficiently random pseudo-random sequences. This fact is not surprising if we think of the sequences in terms of the structures created in Fig. 11 and Fig. 12. The structures have rules that the sequences adhere, i.e. the sequences represent the orientation of the arcs. There are patterns in these structures, and therefore, we can expect, to some extent, the sequences to exhibit patterns as well. Each fractal is not a jumbled mess of random orientations, but it is structured. Note that the sequences are not periodic either as proven in Sec. 1.3.3, for if they were, the value of entropy would be zero.

The results in Table 4 pose some interesting questions. Looking at the arrowhead (Fig. 12(a))and crab (Fig. 12(b))fractals, although they are of different shapes, they have the same complexity. Indeed, our method produces the *same ε*-machines for them, and thus results in the same measure of complexity. This could be explained by the fact that the arc-fractal sequences from the out-in-out (crab) and the in-out-in (arrowhead) constructions are complementary, i.e. the same results can be obtained by “flipping” the rules (out-in-out to in-out-in or vice versa).

Next, we can immediately see that the out-out (Lévy, Fig. 11(a))construction rule possesses mirror symmetry about the vertical direction, whilst the out-in counterpart (Heighway, Fig. 11(b))possesses less prominent symmetry (it has central symmetry, though not exact). Intuitively, something that displays less symmetry should be more complex. But the result shows that Lévy is more complex than Heighway. It is suggestive that there is some information not captured in the sequence. Indeed, the sequence corresponding to an arc-fractal contains the orientation of the arcs, but not the complete spatial information about the arcs, such as their relative positions among each other, or even their locations in space. In this sense, the sequence forms the macroscopic pattern of the arc-fractal, but it does not capture its microscopic rules.

Pertaining to the rules of generation, we can see that the choice of the number of arcs *n* contributes to different number of symbols. In the context of the *ε*-machine, this gives rise to different number of *ε*-machine state configurations. The more possible combinations (of a symbol followed by another) there are, the more states that the *ε*-machine has and, hence, the higher the complexity is. It is, therefore, reasonable to expect that a rule that generates higher number of symbols typically results in higher complexity, which is confirmed by the results. Furthermore, choosing different combinations of orientation ***ω*** may also result in different values of complexity, as shown by Lévy and Heighway fractals. This tells us that the orientations also affect the configuration of states in the *ε*-machine.

As already mentioned, the metric entropy is just a measure of randomness. The measure of complexity as mixture of randomness and regularity tells us more about the pattern of the arc-fractals. From the perspective of the metric entropy, the sequences generally possess low randomness. High degree of complexity indicates that there is a certain extent of regularity or order that pertains to the pattern exhibited by the arc-fractals, and this complexity arises because of the rules of the arc-fractals.

Comparison of measure of complexity between the present arc-fractal sequences and that of the logistic map performed by [12] also reveals further interesting features of the arc-fractal system. Theoretically, we expect the complexity of the edge of chaos of the logistic map to be infinite. However, due to finite implementation, the resulting measure takes finite value numerically [12]. In this context, the symbolic sequence generated by the arc-fractal system is found to attain a higher complexity measure relative to the edge of chaos of the logistic map (at fixed parameter *N* = 16) in consequence of the higher degree of mixture between regularity and randomness, as observed from the generated *ε*-machine. This fact shows that the sequences indeed possess significant level of complexity.

## Conclusion

In conclusion, we have introduced the sequences obtained from a simple *deterministic* system called the arc-fractal system. We have discussed a number of properties of the sequences including the fact that they comprise of only *a few symbols* and are *not periodic*. We then employ the measure of complexity developed earlier in the literature to address the question of complexity of the sequences. The results show that the sequences indeed possess significant level of complexity, which is comparable to that of the known example of logistic map at the edge of chaos reproduced by our implementation in this work.
